# Bis[2,2′-(2-amino­ethyl­imino)­di(ethyl­ammonium)] di-μ-sulfido-bis[disulfido­stannate(IV)]

**DOI:** 10.1107/S1600536811038657

**Published:** 2011-10-12

**Authors:** Emma Karey, Kimberly A. Rosmus, Jennifer A. Aitken, Joseph MacNeil

**Affiliations:** a Department of Chemistry, Chatham University, 1 Woodland Road, Pittsburgh, PA 15232, USA; b Department of Chemistry and Biochemistry, Duquesne University, 600 Forbes Avenue, Pittsburgh, PA 15282, USA

## Abstract

The asymmetric unit of the title compound, (C_6_H_20_N_4_)_2_[Sn_2_S_6_], comprises half of a [Sn_2_S_6_]^4−^ anion and a diprotonated tris­(2-amino­eth­yl)amine cation. The anion lies on an inversion center, while the atoms of the cation occupy general positions. An intra­molecular N—H⋯N hydrogen bond is observed in the cation. In the crystal, strong N—H⋯S hydrogen bonding between the terminal sulfur atoms of the anion and the protonated amine N atoms of the cations result in a three-dimensional network.

## Related literature

For synthetic conditions and the structure of the hydrated form of this complex, see: Näther *et al.* (2003 ▶). For solvothermal syntheses of compounds with [Sn_2_S_6_]^4−^ anions, see: Behrens *et al.* (2003 ▶); Jia *et al.* (2005 ▶); Jiang *et al.* (1998*a* ▶); Li *et al.* (1997 ▶). For other thio­stannate anions, see: Jiang *et al.* (1998*b* ▶). For a review article covering related compounds, see: Zhou *et al.* (2009 ▶). 
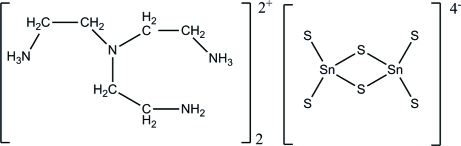


## Experimental

### 

#### Crystal data

(C_6_H_20_N_4_)_2_[Sn_2_S_6_]
*M* *_r_* = 363.13
Monoclinic, 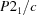

*a* = 9.9280 (2) Å
*b* = 14.8845 (3) Å
*c* = 10.2498 (2) Å
β = 115.758 (1)°
*V* = 1364.15 (5) Å^3^
*Z* = 4
Mo *K*α radiation
μ = 2.31 mm^−1^
*T* = 296 K
0.61 × 0.57 × 0.39 mm

#### Data collection

Bruker SMART APEX diffractometer
Absorption correction: multi-scan (*SADABS*; Sheldrick, 2002 ▶) *T*_min_ = 0.334, *T*_max_ = 0.467
25432 measured reflections
4907 independent reflections
4460 reflections with *I* > 2σ(*I*)
*R*_int_ = 0.023

#### Refinement

*R*[*F* ^2^ > 2σ(*F* ^2^)] = 0.021
*wR*(*F* ^2^) = 0.058
*S* = 1.04
4907 reflections
132 parameters
H-atom parameters constrained
Δρ_max_ = 1.22 e Å^−3^
Δρ_min_ = −0.51 e Å^−3^

### 

Data collection: *SMART* (Bruker, 1998 ▶); cell refinement: *SAINT* (Bruker, 1998 ▶); data reduction: *SAINT*; program(s) used to solve structure: *SHELXS97* (Sheldrick, 2008 ▶); program(s) used to refine structure: *SHELXL97* (Sheldrick, 2008 ▶); molecular graphics: *CrystalMaker* (Palmer, 2010 ▶); software used to prepare material for publication: *publCIF* (Westrip, 2010 ▶).

## Supplementary Material

Crystal structure: contains datablock(s) I, New_Global_Publ_Block. DOI: 10.1107/S1600536811038657/si2373sup1.cif

Structure factors: contains datablock(s) I. DOI: 10.1107/S1600536811038657/si2373Isup2.hkl

Additional supplementary materials:  crystallographic information; 3D view; checkCIF report

## Figures and Tables

**Table 1:** Selected bond lengths (Å)

Sn1—S1	2.3307 (4)
Sn1—S2	2.3447 (4)
Sn1—S3^i^	2.4550 (4)
Sn1—S3	2.4564 (4)

Symmetry code: (i) 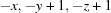.

**Table 2:** Hydrogen-bond geometry (Å, °)

*D*—H⋯*A*	*D*—H	H⋯*A*	*D*⋯*A*	*D*—H⋯*A*
N9—H9*C*⋯N10	0.89	2.10	2.965 (3)	163
N9—H9*B*⋯S1^ii^	0.89	2.44	3.314 (2)	167
N9—H9*A*⋯S2^iii^	0.89	2.49	3.370 (2)	168
N7—H7*C*⋯S1^i^	0.89	2.36	3.243 (2)	174
N7—H7*B*⋯S2^iv^	0.89	2.40	3.278 (2)	170
N7—H7*A*⋯S2^v^	0.89	2.57	3.411 (2)	159

Symmetry codes: (i) 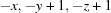; (ii) 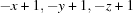; (iii) 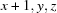; (iv) 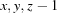; (v) 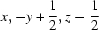.

**Fig. 1.**
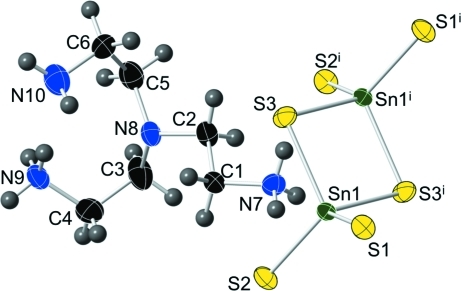
The structure of (C6H20N4)2[Sn2S6].The thermal ellipsoids have been drawn at the 50% probability level. Symmetry code: (i) = -x, -y + 1, -z + 1.

**Fig. 2.**
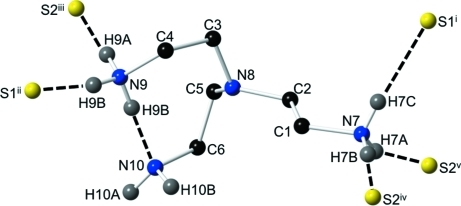
Hydrogen bonding interactions within the (H2tren)2+ cation and between the cation and the terminal sulfurs of the [Sn2S6]4- anion. Symmetry codes as presented in Table 2.

**Fig. 3.**
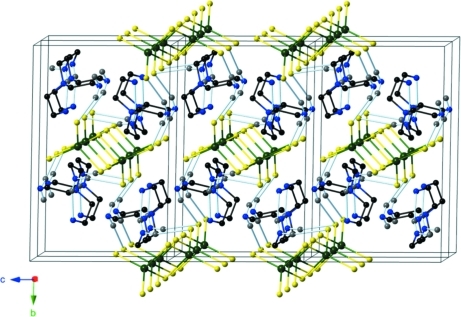
View down the a axis illustrating the three-dimensional hydrogen bonding network.

## supplementary crystallographic information

### Comment

While solvothermal syntheses have produced an assortment of anionic thiostannate building blocks, the [Sn_2_S_6_]^4-^ moiety has been one of the most common (Zhou *et al.*, 2009). It has also been shown that under certain conditions [Sn_2_S_6_]^4-^ anions can be converted to forms such as [Sn_3_S_7_]^2-^ and [Sn_4_S_9_]^-^, forming two-dimensional layered anionic networks (Jiang *et al.*, 1998*a*,*b*). In 2003, solvothermal experiments aimed at preparing [Co(tren)_2_][Sn_2_S_6_] using the chelating ligand tris(2-aminoethyl)amine (C_6_H_18_N_4_, tren), resulted in the isolation and characterization of (H_2_tren)_2_[Sn_2_S_6_]^.^2H_2_O (Näther *et al.*, 2003) as a side product. We have now shown that the anhydrous version of this compound, (I) can be accessed when the reaction is carried out in anhydrous conditions and without any transition metal present. In the title compound, (H_2_tren)_2_[Sn_2_S_6_], (Fig. 1), the terminal Sn—S bonds, at 2.3307 (4) and 2.3447 (5) Å, are shorter than the Sn—S bond of 2.4565 (6) Å formed with the bridging sulfur. The interior S—Sn—S angle of 92.78 (2)° is tighter than those involving terminal sulfurs, where these angles range from 108.22 (2) to 120.55 (2)°. Collectively, the geometric parameters of the [Sn_2_S_6_]^4-^ anion are in reasonable accordance with similar structures (Näther *et al.*, 2003; Behrens *et al.*, 2003). In the (H_2_tren)^2+^ cation, the C—N bond lengths for the two protonated pendant amines are slightly longer, at 1.490 (3) and 1.473 (3) Å, than the 1.446 (5) Å C—N bond for the neutral arm. The most distinct structural difference between the anhydrous structure and the previously reported hydrated form (Näther *et al.*, 2003) is the positioning of the NH_2_ pedant amine. In the hydrated structure, it is aligned to facilitate a H-bonding interaction (H···N—H of 2.08 Å) with an NH_3_^+^ amine on a neighboring cation. In the anhydrous structure, the hydrogen bonding interaction (N9—H9C···N10, 2.10 Å) is formed within the same ligand, Fig. 2. Strong hydrogen bonding between the terminal sulfur atoms on the anion and the protonated amine centers on the cation (Fig. 2, Table 2) results in a three-dimensional network, Fig. 3.

### Experimental

The title compound was prepared by solvothermal synthesis, using conditions comparable to Näther *et al.* (2003). 5.0 ml of tris-2-aminoethylamine (tren) was mixed with 1.00 mmol Sn and 3.0 mmol S in a 23 ml Parr^(^*^R^*^)^; acid digestion apparatus. The mixture was heated to 423 K over 5 h and maintained at that temperature for 144 h. It was cooled to 363 K at 2 K/h, then cooled to 313 K at 6 K/h. The clear, colorless crystals were washed with hexane and recovered by vacuum filtration. This protocol produced large crystals, often several mm on the longest axis, and in one instance measuring over 20 mm.

### Refinement

All H atoms except for those on N(10) were placed at calculated positions (C—H at 0.97 Å, N—H at 0.87 Å) and refined as riding atoms with *U*_iso_(H) = 1.2*U*_eq_(C) and *U*_iso_(H) = 1.5*U*_eq_(N). The two H atoms on N(10) were identified from the difference Fourier map and refined as a rigid group with *U*_iso_(H) = 1.5*U*_eq_(N).

### Crystal data

**Table:**

(C_6_H_20_N_4_)_2_[Sn_2_S_6_]	*F*(000) = 728
*M**_r_* = 363.13	*D*_x_ = 1.768 Mg m^−^^3^
Monoclinic, *P*2_1_/*c*	Mo *K*α radiation, λ = 0.71073 Å
Hall symbol: -P 2ybc	Cell parameters from 9995 reflections
*a* = 9.9280 (2) Å	θ = 2.3–33.1°
*b* = 14.8845 (3) Å	µ = 2.31 mm^−^^1^
*c* = 10.2498 (2) Å	*T* = 296 K
β = 115.758 (1)°	Clear, colourless
*V* = 1364.15 (5) Å^3^	0.61 × 0.57 × 0.39 mm
*Z* = 4	

### Data collection

**Table:**

Bruker SMART APEX diffractometer	4907 independent reflections
Radiation source: fine-focus sealed tube	4460 reflections with *I* > 2σ(*I*)
graphite	*R*_int_ = 0.023
φ and ω Scans scans	θ_max_ = 33.1°, θ_min_ = 2.3°
Absorption correction: multi-scan (*SADABS*; Sheldrick, 2002)	*h* = −14→14
*T*_min_ = 0.334, *T*_max_ = 0.467	*k* = −22→22
25432 measured reflections	*l* = −15→15

### Refinement

**Table:**

Refinement on *F*^2^	Primary atom site location: structure-invariant direct methods
Least-squares matrix: full	Secondary atom site location: difference Fourier map
*R*[*F*^2^ > 2σ(*F*^2^)] = 0.021	Hydrogen site location: inferred from neighbouring sites
*wR*(*F*^2^) = 0.058	H-atom parameters constrained
*S* = 1.04	*w* = 1/[σ^2^(*F*_o_^2^) + (0.0298*P*)^2^ + 0.7916*P*] where *P* = (*F*_o_^2^ + 2*F*_c_^2^)/3
4907 reflections	(Δ/σ)_max_ = 0.002
132 parameters	Δρ_max_ = 1.22 e Å^−^^3^
0 restraints	Δρ_min_ = −0.51 e Å^−^^3^

### Special details

**Table:**

Geometry. All e.s.d.'s (except the e.s.d. in the dihedral angle between two l.s. planes) are estimated using the full covariance matrix. The cell e.s.d.'s are taken into account individually in the estimation of e.s.d.'s in distances, angles and torsion angles; correlations between e.s.d.'s in cell parameters are only used when they are defined by crystal symmetry. An approximate (isotropic) treatment of cell e.s.d.'s is used for estimating e.s.d.'s involving l.s. planes.
Refinement. Refinement of *F*^2^ against ALL reflections. The weighted *R*-factor *wR* and goodness of fit *S* are based on *F*^2^, conventional *R*-factors *R* are based on *F*, with *F* set to zero for negative *F*^2^. The threshold expression of *F*^2^ > σ(*F*^2^) is used only for calculating *R*-factors(gt) *etc*. and is not relevant to the choice of reflections for refinement. *R*-factors based on *F*^2^ are statistically about twice as large as those based on *F*, and *R*- factors based on ALL data will be even larger.

### Fractional atomic coordinates and isotropic or equivalent isotropic displacement parameters (Å^2^)

**Table:**

	*x*	*y*	*z*	*U*_iso_*/*U*_eq_	
Sn1	0.01082 (1)	0.468130 (7)	0.66292 (1)	0.02312 (4)	
S1	0.18598 (5)	0.52916 (3)	0.87984 (5)	0.03186 (9)	
S2	−0.15015 (5)	0.35310 (3)	0.66377 (5)	0.03259 (9)	
S3	0.13815 (5)	0.41901 (3)	0.51649 (5)	0.03229 (9)	
C1	0.1339 (2)	0.3225 (1)	0.0717 (2)	0.0349 (3)	
H1A	0.1600	0.3833	0.0573	0.042*	
H1B	0.1715	0.2818	0.0213	0.042*	
C2	0.2059 (2)	0.3005 (1)	0.2327 (2)	0.0380 (4)	
H2A	0.1620	0.3379	0.2817	0.046*	
H2B	0.1862	0.2382	0.2463	0.046*	
C3	0.4166 (3)	0.4070 (2)	0.3308 (4)	0.0552 (6)	
H3A	0.4711	0.4130	0.4351	0.066*	
H3B	0.3308	0.4468	0.2984	0.066*	
C4	0.5166 (3)	0.4348 (2)	0.2610 (3)	0.0528 (6)	
H4A	0.4596	0.4337	0.1565	0.063*	
H4B	0.5507	0.4959	0.2895	0.063*	
C5	0.4481 (3)	0.2526 (2)	0.4178 (2)	0.0466 (5)	
H5A	0.5397	0.2814	0.4852	0.056*	
H5B	0.3875	0.2407	0.4689	0.056*	
C6	0.4856 (3)	0.1665 (2)	0.3699 (3)	0.0510 (5)	
H6A	0.5435	0.1297	0.4538	0.061*	
H6B	0.3942	0.1344	0.3106	0.061*	
N7	−0.0318 (2)	0.3140 (1)	0.0112 (2)	0.0350 (3)	
H7A	−0.0551	0.2610	0.0361	0.053*	
H7B	−0.0715	0.3181	−0.0849	0.053*	
H7C	−0.0677	0.3577	0.0462	0.053*	
N8	0.3655 (2)	0.3154 (1)	0.2955 (2)	0.0340 (3)	
N9	0.6473 (2)	0.3752 (1)	0.3030 (2)	0.0393 (4)	
H9A	0.7035	0.3791	0.3981	0.059*	
H9B	0.7009	0.3916	0.2564	0.059*	
H9C	0.6165	0.3187	0.2799	0.059*	
N10	0.5705 (3)	0.1814 (1)	0.2875 (3)	0.0535 (5)	
H10A	0.6363	0.1388	0.2887	0.080*	
H10B	0.5046	0.1868	0.1857	0.080*	

### Atomic displacement parameters (Å^2^)

**Table:**

	*U*^11^	*U*^22^	*U*^33^	*U*^12^	*U*^13^	*U*^23^
Sn1	0.02520 (5)	0.02399 (6)	0.02090 (5)	−0.00209 (3)	0.01070 (4)	−0.00039 (3)
S1	0.0316 (2)	0.0363 (2)	0.0254 (2)	−0.0069 (1)	0.0102 (1)	−0.0050 (1)
S2	0.0325 (2)	0.0375 (2)	0.0268 (2)	−0.0115 (2)	0.0120 (1)	0.0009 (1)
S3	0.0400 (2)	0.0321 (2)	0.0300 (2)	0.0127 (2)	0.0201 (2)	0.0057 (1)
C1	0.0357 (8)	0.0370 (9)	0.0362 (9)	0.0016 (7)	0.0196 (7)	0.0056 (7)
C2	0.0316 (8)	0.049 (1)	0.0347 (9)	−0.0045 (7)	0.0157 (7)	−0.0022 (8)
C3	0.052 (1)	0.036 (1)	0.089 (2)	−0.0123 (9)	0.041 (1)	−0.019 (1)
C4	0.049 (1)	0.037 (1)	0.075 (2)	−0.0066 (8)	0.022 (1)	0.011 (1)
C5	0.042 (1)	0.062 (1)	0.037 (1)	−0.0056 (9)	0.0190 (9)	−0.0023 (9)
C6	0.043 (1)	0.056 (1)	0.053 (1)	0.0121 (9)	0.020 (1)	0.021 (1)
N7	0.0356 (7)	0.0375 (8)	0.0291 (7)	0.0014 (6)	0.0112 (6)	0.0019 (6)
N8	0.0280 (6)	0.0343 (7)	0.0407 (8)	−0.0060 (5)	0.0160 (6)	−0.0056 (6)
N9	0.0330 (7)	0.0461 (9)	0.0379 (8)	−0.0106 (7)	0.0146 (6)	0.0023 (7)
N10	0.055 (1)	0.049 (1)	0.067 (1)	−0.0051 (9)	0.037 (1)	−0.009 (1)

### Geometric parameters (Å, °)

**Table:**

Sn1—S1	2.3307 (4)	C4—H4A	0.9700
Sn1—S2	2.3447 (4)	C4—H4B	0.9700
Sn1—S3^i^	2.4550 (4)	C5—C6	1.477 (4)
Sn1—S3	2.4564 (4)	C5—N8	1.491 (3)
S3—Sn1^i^	2.4550 (4)	C5—H5A	0.9700
C1—N7	1.490 (2)	C5—H5B	0.9700
C1—C2	1.521 (3)	C6—N10	1.446 (3)
C1—H1A	0.9700	C6—H6A	0.9700
C1—H1B	0.9700	C6—H6B	0.9700
C2—N8	1.445 (2)	N7—H7A	0.8900
C2—H2A	0.9700	N7—H7B	0.8900
C2—H2B	0.9700	N7—H7C	0.8900
C3—N8	1.444 (3)	N9—H9A	0.8900
C3—C4	1.512 (4)	N9—H9B	0.8900
C3—H3A	0.9700	N9—H9C	0.8900
C3—H3B	0.9700	N10—H10A	0.9066
C4—N9	1.474 (3)	N10—H10B	0.9645
			
S1—Sn1—S2	120.55 (2)	C6—C5—N8	113.0 (2)
S1—Sn1—S3^i^	113.87 (2)	C6—C5—H5A	109.0
S2—Sn1—S3^i^	108.22 (2)	N8—C5—H5A	109.0
S1—Sn1—S3	109.28 (2)	C6—C5—H5B	109.0
S2—Sn1—S3	108.51 (2)	N8—C5—H5B	109.0
S3^i^—Sn1—S3	92.78 (1)	H5A—C5—H5B	107.8
Sn1^i^—S3—Sn1	87.22 (1)	N10—C6—C5	110.8 (2)
N7—C1—C2	110.3 (1)	N10—C6—H6A	109.5
N7—C1—H1A	109.6	C5—C6—H6A	109.5
C2—C1—H1A	109.6	N10—C6—H6B	109.5
N7—C1—H1B	109.6	C5—C6—H6B	109.5
C2—C1—H1B	109.6	H6A—C6—H6B	108.1
H1A—C1—H1B	108.1	C1—N7—H7A	109.5
N8—C2—C1	110.9 (2)	C1—N7—H7B	109.5
N8—C2—H2A	109.5	H7A—N7—H7B	109.5
C1—C2—H2A	109.5	C1—N7—H7C	109.5
N8—C2—H2B	109.5	H7A—N7—H7C	109.5
C1—C2—H2B	109.5	H7B—N7—H7C	109.5
H2A—C2—H2B	108.0	C2—N8—C3	117.1 (2)
N8—C3—C4	111.8 (2)	C2—N8—C5	112.0 (2)
N8—C3—H3A	109.3	C3—N8—C5	112.1 (2)
C4—C3—H3A	109.3	C4—N9—H9A	109.5
N8—C3—H3B	109.3	C4—N9—H9B	109.5
C4—C3—H3B	109.3	H9A—N9—H9B	109.5
H3A—C3—H3B	107.9	C4—N9—H9C	109.5
N9—C4—C3	111.9 (2)	H9A—N9—H9C	109.5
N9—C4—H4A	109.2	H9B—N9—H9C	109.5
C3—C4—H4A	109.2	C6—N10—H10A	119.0
N9—C4—H4B	109.2	C6—N10—H10B	110.6
C3—C4—H4B	109.2	H10A—N10—H10B	102.6
H4A—C4—H4B	107.9		

Symmetry codes: (i) −*x*, −*y*+1, −*z*+1.

### Hydrogen-bond geometry (Å, °)

**Table:**

*D*—H···*A*	*D*—H	H···*A*	*D*···*A*	*D*—H···*A*
N9—H9C···N10	0.89	2.10	2.965 (3)	163.
N9—H9B···S1^ii^	0.89	2.44	3.314 (2)	167.
N9—H9A···S2^iii^	0.89	2.49	3.370 (2)	168.
N7—H7C···S1^i^	0.89	2.36	3.243 (2)	174.
N7—H7B···S2^iv^	0.89	2.40	3.278 (2)	170.
N7—H7A···S2^v^	0.89	2.57	3.411 (2)	159.

Symmetry codes: (ii) −*x*+1, −*y*+1, −*z*+1; (iii) *x*+1, *y*, *z*; (i) −*x*, −*y*+1, −*z*+1; (iv) *x*, *y*, *z*−1; (v) *x*, −*y*+1/2, *z*−1/2.
